# Self-Regulated Symmetry Breaking Model for Stem Cell Differentiation

**DOI:** 10.3390/e25050815

**Published:** 2023-05-18

**Authors:** Madelynn McElroy, Kaylie Green, Nikolaos K. Voulgarakis

**Affiliations:** 1 Department of Mathematics and Statistics, Washington State University, Pullman, WA 99164, USA; madelynn.mcelroy@wsu.edu (M.M.); kaylie.j.green@wsu.edu (K.G.); 2 Voiland School of Chemical Engineering and Bioengineering, Washington State University, Pullman, WA 99164, USA

**Keywords:** cell differentiation, phase transitions, symmetry breaking, self-tuned criticality, mean field theory, bifurcation theory

## Abstract

In conventional disorder–order phase transitions, a system shifts from a highly symmetric state, where all states are equally accessible (disorder) to a less symmetric state with a limited number of available states (order). This transition may occur by varying a control parameter that represents the intrinsic noise of the system. It has been suggested that stem cell differentiation can be considered as a sequence of such symmetry-breaking events. Pluripotent stem cells, with their capacity to develop into any specialized cell type, are considered highly symmetric systems. In contrast, differentiated cells have lower symmetry, as they can only carry out a limited number of functions. For this hypothesis to be valid, differentiation should emerge collectively in stem cell populations. Additionally, such populations must have the ability to self-regulate intrinsic noise and navigate through a critical point where spontaneous symmetry breaking (differentiation) occurs. This study presents a mean-field model for stem cell populations that considers the interplay of cell–cell cooperativity, cell-to-cell variability, and finite-size effects. By introducing a feedback mechanism to control intrinsic noise, the model can self-tune through different bifurcation points, facilitating spontaneous symmetry breaking. Standard stability analysis showed that the system can potentially differentiate into several cell types mathematically expressed as stable nodes and limit cycles. The existence of a Hopf bifurcation in our model is discussed in light of stem cell differentiation.

## 1. Introduction

In the course of early embryonic development, a group of identical stem cells actively self-replicates while simultaneously differentiating into all the specialized cells necessary for the formation of a multicellular organism. Although development progresses with remarkable precision, small populations of stem cells display significant cell-to-cell variability due to the molecular nature of gene expression [1,2]. It is truly remarkable how this seemingly fragile and heterogeneous cellular mass transforms into a multicellular organism with such exceptional accuracy.

Waddington envisioned the differentiation process as analogous to a ball rolling down a hillside of a complex landscape containing various slopes and valleys [3]. In this picture, each valley represents a possible state of the cell during development. Stem cells start at the top of the hill, where they are in a relatively undifferentiated state. The ball, which represents the stem cell, starts rolling down the hill, passing through different slopes and valleys. The path the ball takes is determined by a combination of intrinsic and extrinsic factors that push it in a particular direction. As the ball rolls, it becomes increasingly specialized (differentiated) until it reaches its final, stable state at the bottom of the hill, where it becomes a committed cell. Even though Waddington’s epigenetic landscape was initially proposed as a metaphor, it has since become a valuable framework for understanding how the gene regulatory network of cells and environmental factors interact to shape the development of organisms.

The present work is built on three major hypotheses that have been proposed over the years and are based on or inspired by Waddington’s epigenetic landscape. First, Huang and collaborators suggested that pluripotent stem cells can simultaneously express multiple genes that are specific to the progenitors [4,5]. Intrinsic and environmental noise can disrupt this state and cause the cell to express a limited number of those genes that correspond to a certain progenitor. In this view, stem cells are considered highly symmetrical cellular states as they can co-express a higher number of genes. In contrast, progenitor cells are considered to operate in a lower symmetry cellular state, as they express a smaller number of genes. This process of symmetry breaking may also occur during the differentiation of progenitor cells into specialized cells. The view of differentiation as a series of symmetry-breaking events is supported by experimental findings in hematopoietic stem cells, for example, which express genes common to all the cell types they can differentiate into [6,7]. This hypothesis indicates that the process of reaching a terminal cell fate is characterized by a decrease in entropy [8]. However, this decrease is preceded by an initial burst-like increase in entropy, which occurs before the differentiation events [8,9]. The crucial question that needs to be addressed is how cells regulate transcriptional noise to drive the system toward a state of lower entropy. This brings us to the second hypothesis, which suggests the existence of intrinsic molecular devices that regulate transcriptional noise [10,11]. Experimental evidence supports this viewpoint, showing that noise is indeed regulated in embryonic stem cells via molecular signaling and feedback mechanisms [10,12]. The third major hypothesis is that pluripotency may arise *collectively* within populations of stem cells through intercellular interactions, such as paracrine and autocrine signaling [13,14,15,16,17]. This hypothesis is primarily supported by experimental evidence indicating that paracrine signaling plays a crucial role in cell differentiation [18,19]. It is also supported by the emergence of a dynamic equilibrium of cell states that is observed only in stem cell populations [20].

The above hypotheses can be interpreted in standard statistical mechanics language as follows. The process of differentiation involves a collection of interacting agents (cells) that undergoes a transition from a highly symmetrical state (pluripotency) to a state of low symmetry (committed cells). This transition is achieved by decreasing the inherent noise within the system. This scenario exhibits similarities to symmetry breaking in phase transitions. Indeed, in traditional disorder–order phase transitions, a system shifts from a state of high entropy or disorder to a state of lower entropy or order by adjusting a control parameter such as temperature, pressure, or an external field [21]. The transition from a paramagnetic to a ferromagnetic state in a magnetic material is a classic example of a disorder–order phase transition. At high temperatures, strong thermal fluctuations force the system into a disordered state of zero magnetization, where each spin points in any possible direction (high symmetry). As the temperature of the magnet is lowered, all molecular spins start pointing in the same direction (low symmetry), providing the system with a nonzero net magnetization. Other examples of disorder–order phase transitions include the transition from a liquid to a solid.

Various methods can be employed to test the validity of these hypotheses. For instance, if the differentiation of stem cells into progenitor cells represents a first-order phase transition, we would expect to observe the coexistence of both cell types at a critical intrinsic noise level. Alternatively, if this process is a second-order phase transition, significant spatiotemporal fluctuations and correlations may be detected when passing through the tipping point. Although the experimental evidence is currently challenging to directly relate to phase transitions, a recent study showed that an increase in both cell heterogeneity and coordination of gene expression occurred prior to induced pluripotent stem cell differentiation [22]. It has to be noted that the analytical theory of phase transitions is applicable only in the thermodynamic limit (infinitely large systems). In this limit, the system experiences strong ergodicity breaking, which stabilizes a long-range ordered state. However, in the case of small systems of interacting agents, such as stem cell populations, finite-size effects cause significant fluctuations in the system’s state. This type of fluctuation decreases with increasing system size. Thus, the observed cell-to-cell variability in stem cell populations could be attributed to a combination of intrinsic noise, finite-size effects, and the possibility of the population undergoing a phase transition.

At the single-cell level, stem cell differentiation has primarily been studied through the lens of ordinary differential equations (ODEs), which display various types of bifurcations, such as supercritical pitchfork and saddle-node bifurcation [23,24]. In this framework, each stable branch in the bifurcation diagram represents a different type of cell, and transitions from one stable branch to another signify cell differentiation. It is possible to model the collective dynamics of stem cell populations by coupling such dynamical systems together through appropriate cell–cell interactions. A prominent example is the work by Furusawa and Kaneko, where they emphasized the significance of oscillatory expression dynamics for preserving an undifferentiated state [25]. In a different study, Huang and colleagues showed that gene regulatory motifs with mutual inhibitory interactions and self-activation can generate multiple attractors corresponding to stem and differentiated states [26]. Other studies have demonstrated that coupled systems of ODEs undergo bifurcations as a function of population size and cell–cell interactions, which occur solely at the population level [27,28,29]. These bifurcations lead to cell differentiation through spontaneous symmetry-breaking events. Similar collective dynamical states have also been observed in experimental settings [30,31,32,33,34].

An alternative method of modeling the dynamics of individual cells is through the use of Boolean networks (BNs) [35,36,37,38]. In BNs, molecular regulators are represented as binary nodes that can exist in an “on” (active) or “off” (inactive) state. These nodes interact with neighboring nodes according to predefined deterministic rules, resulting in various attractors that represent possible functions of the cell. Recently, we implemented coupled BNs to describe the collective dynamics of stem cell populations [39]. In this study, individual cell dynamics were described by isogenic BNs, while a multilayer Ising Hamiltonian was used to model cell–cell interaction through paracrine and autocrine signaling. Numerical analysis in two dimensions revealed that changes in intrinsic noise can induce a series of spontaneous symmetry-breaking events, resulting in various types of cells. In our model, pluripotency is defined as the state of a balanced mixture of next-generation cells (progenitors). When the intrinsic noise drops below a critical threshold, the system undergoes symmetry breaking, leading the population to assume a specific cell state (differentiation). Similar to experimental observations [22], prior to cell differentiation, our model exhibited significant cell-to-cell variability. This type of symmetry breaking resembles the disorder–order phase transition in statistical mechanics.

While coupled ODEs and coupled BNs have distinct technical and theoretical characteristics, both can effectively model the collective behavior of stem cell populations. However, coupled BNs that use an Ising-type Hamiltonian offer a unique opportunity to investigate the applicability of phase transitions in stem cell populations. For instance, recent experimental observations of increased cell heterogeneity and the spatiotemporal correlation of gene expression [22] can be qualitatively described by our coupled BN model [39]. Here, we aimed to simplify our previous work by implementing Landau’s mean-field theory of phase transitions (see, for example, [40]). Although this approach is a crude approximation of the collective dynamics of coupled BNs, it provides a simpler way to understand the underlying phenomenology of noise-regulated cell differentiation. Here, we hypothesize that the noise regulation depends on the current state of the population and is governed by a negative feedback equation that considers the interplay between cell cooperativity and heterogeneity. This methodology led to several compelling outcomes, including the existence of diverse types of attractors (cell types). As also predicted by Landau’s original theory, our dynamical system exhibits hysteresis loops that could potentially model phenotypic memory. To address the impact of finite-size effects in small stem cell populations, we incorporated a stochastic forcing term that decreases proportionally with system size. Notably, finite-size effects can cause intriguing phenomena, such as noise-induced bistability and overall phenotype switching [41,42,43,44].

The paper’s structure is straightforward. In Section 2, we examine Landau’s potential function and briefly overview its bifurcation and phase transition analysis. We subsequently introduce the feedback mechanism that self-regulates the system’s intrinsic noise. A standard way of incorporating finite-size effects is also discussed. In Section 3, we analyze the stability of our model and examine the impact of self-regulation and finite-size effects on the attractors’ properties. Finally, Section 4 summarizes our results.

## 2. Mean-Field Approximation Model for Self-Tuned Symmetry Breaking

### 2.1. Landau’s Potential Energy for Stem Cell Populations

In our recent study [39], we utilized a system of coupled BNs to model the collective dynamics of stem cell populations. Through numerical analysis, we discovered that, by adjusting the level of intrinsic noise, the system undergoes a series of symmetry-breaking events that can be interpreted as cell differentiation. Interestingly, these symmetry-breaking events exhibit characteristics similar to order–disorder phase transitions in statistical mechanics. Here, we aimed to simplify our model by implementing Landau’s phenomenology of phase transitions. In the mean-field approximation, the order parameter *m* evolves under the influence of the following dimensionless potential function:

(1) $$V=-\frac{1-\xi }{2}m^{2}+\frac{\alpha }{3}m^{3}+\frac{\beta }{4}m^{4}+\frac{\gamma }{6}m^{6},$$

where ξ≥0 is the intrinsic noise of the system and α,γ∈{0,1}, and β∈{−1,1}. The time evolution of *m* is dictated by $\dot{m}=-dV/dm$. This approach was originally developed to explain order–disorder phase transitions that occur by varying the temperature (ξ) of systems. In such systems, m=0 signifies a disordered state, while m≠0 describes ordered states. As the temperature is lowered below a critical point, the system undergoes a phase transition from a state of high symmetry (disorder) to a state of lower symmetry (order). Note that this approach is applicable only in the thermodynamic limit, i.e., infinitely large systems. In the case of finite systems, it is possible to assume that the system is driven by the same potential function, but is subject to random fluctuations that are inversely proportional to the size of the system, *N*. In this approximation, the time evolution of the order parameter is given by the following stochastic differential equation:

(2) $$dm_{t}=\left[\left(1-\xi \right)m_{t}-\alpha m_{t}^{2}-\beta m_{t}^{3}-\gamma m_{t}^{5}\right]dt+\sigma dW_{t},$$

where *t* represents time, $\sigma ∼1\sqrt{N}$ is the standard deviation of the random fluctuations, and $W_{t}$ is the standard Brownian motion. In the thermodynamic limit, where σ→0, the differential Equation (2) can be studied through the lens of bifurcation or phase transition theory. A bifurcation is described as a sudden change in the stability of the system; either new equilibrium states emerge, or the stability of existing states changes. On the other hand, a phase transition occurs when the global minimum of the potential function (ground state) shifts from one state to another. This shift may be continuous (second-order phase transition) or discontinuous (first-order phase transition) [21]. As we will see below, the bifurcation and phase transition diagrams may not necessarily coincide.

In this work, we drew the analogy from traditional phase transitions and allowed ξ to represent the strength of gene expression noise and *m* to define the average cell type of stem cell populations. Specifically, we considered the case where m=0 corresponds to pluripotent stem cells, while m≠0 describes differentiated cells. In what follows, we used $m_{0}$ to denote the pluripotent state. Let us briefly discuss the results of three distinct models through the prism of this analogy [40]. The bifurcation and phase transition analysis presented below is based on four critical values of the intrinsic noise, $\xi_{c}=1$, $\xi_{c_{1}}=1.18$, $\xi_{c_{2}}=1.22$, and $\xi_{c_{3}}=1.25$.

**Figure 1:**
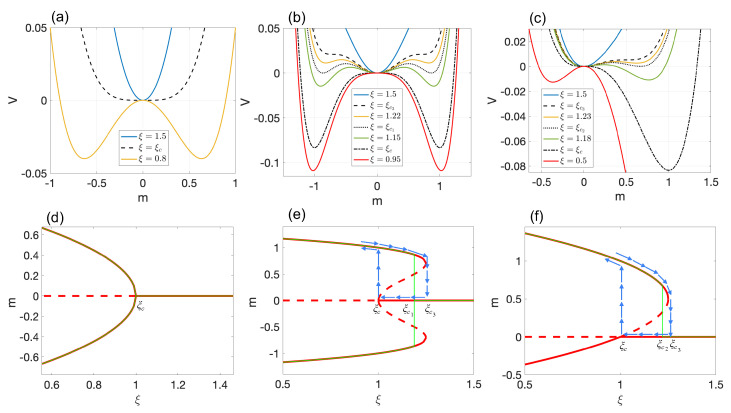
First row: Potential function vs. *m* for (**a**) Model 1, (**b**) Model 2, and (**c**) Model 3. Black lines correspond to critical values of intrinsic noise. Second row: Bifurcation and phase transition diagrams for (**d**) Model 1, (**e**) Model 2, and (**f**) Model 3. Red lines correspond to stable (solid line) and unstable (dashed line) equilibrium points. Green lines indicate phase transitions. Blue arrows demonstrate hysteresis loops. Specifically: (**d**) A supercritical pitchfork bifurcation and a second-order phase transition occur at $\xi_{c}$. (**e**) A subcritical pitchfork and double−saddle−node bifurcation occurs at $\xi_{c}$ and $\xi_{c3}$, respectively. At $\xi_{c_{1}}$, we have a first-order phase transition. (**f**) A transcritical and single-saddle-node bifurcation occur at $\xi_{c}$ and $\xi_{c3}$, respectively. At $\xi_{c_{2}}$, we observe a first-order phase transition.

*Model 1*: Here, we set α=0, β=1, and γ=0. For $\xi \geq \xi_{c}$, the pluripotent cell state ($m_{0}$) is both stable and the ground state of the potential function (solid blue line in Figure 1a). Below the critical noise $\xi_{c}$, the pluripotent state becomes unstable, and two new stable cell states, $m_{\pm }=\pm \sqrt{1-\xi }$, with the same energy emerge (solid yellow line in Figure 1a). Since both new cell types are energetically equally favorable, the ultimate cell fate is decided spontaneously. The change of *m* as a function of ξ is continuous, and as a result, new cell states are reversible under moderate changes in intrinsic noise [23]. This bifurcation class is called supercritical pitchfork bifurcation, and the phase transition is of second order. In this case, the bifurcation and phase transition diagrams coincide (see Figure 1d).

*Model 2*: In this model, α=0, β=−1, and γ=1. The bifurcation analysis of this model is based on $\xi_{c}$ and $\xi_{c_{3}}$. The behavior of the potential function is presented in Figure 1b. For $\xi >\xi_{c_{3}}$, only the pluripotent cell state, $m_{0}$, exists (solid blue line). At $\xi =\xi_{c_{3}}$, the system undergoes a double-saddle-node bifurcation (black dashed line). For $\xi <\xi_{c}$, two new stable $m_{\pm }=\pm \sqrt{0.5+\sqrt{1.25-\xi }}$ and two unstable $\mu_{\pm }=\pm \sqrt{0.5-\sqrt{1.25-\xi }}$ cell types emerge (solid yellow line). By reducing the noise below $\xi_{c}$, $\mu_{\pm }$ disappear, and the pluripotent state $m_{0}$ becomes unstable (red solid line). The complete bifurcation diagram is presented in Figure 1e. Interestingly, this type of model exhibits a hysteresis loop that may be attributed to phenotypic memory (see blue arrows in Figure 1e). In other words, new cell type formation is usually irreversible under moderate changes of intrinsic noise [23].

In this model, the ground state of the system shifts from m=0 to $m_{\pm }$ at $\xi_{c_{1}}$ (dotted black line in Figure 1b). This shifting occurs discontinuously, and thus, the phase transition is of the first order. At this critical point, the pluripotent and differentiated cell states co-exist. Since both $m_{\pm }$ are energetically equally favorable, the differentiation process is spontaneous in this case as well. This model belongs to the saddle-node bifurcation class and is accompanied by a first-order phase transition. As seen in Figure 1d, the bifurcation and phase transition diagrams are notably different. One may say that first-order phase transitions are preceded by saddle-node bifurcations.

*Model 3*: This model utilizes the values of α=−1, β=1, and γ=0. The bifurcation analysis is based on the same critical values of intrinsic noise as in Model 2. Similar to the previous two models, several plots of the potential energy are presented in Figure 1c. When ξ exceeds $\xi_{c_{3}}$, only the pluripotent state, $m_{0}$, exists (solid blue line). At $\xi =\xi_{c_{3}}$, the system undergoes a single-saddle-node bifurcation, resulting in the emergence of two equilibrium points $m_{\pm }=0.5\pm \sqrt{1.25-\xi }$ (dashed black line). Among the two, only $m_{+}$ is stable, indicating the emergence of a new cell type. Decreasing the intrinsic noise at $\xi_{c}$ triggers a transcritical bifurcation, causing the stability of $m_{0}$ and $m_{-}$ to switch (dashed–dotted black line). When ξ falls below $\xi_{c}$, both $m_{\pm }$ become stable, leading to the emergence of two new cell types (solid red line). The complete bifurcation diagram is presented in Figure 1f.

In this model, the ground state of the system shifts discontinuously from $m_{0}$ to $m_{+}$ at a critical point of $\xi_{c_{2}}$ (first-order phase transition). Even after $m_{-}$ becomes stable, $m_{+}$ is always the energetically most-favorable. Notably, this type of model exhibits a hysteresis loop only for $m_{+}$ (see blue arrows in Figure 1f). Thus, the differentiation of $m_{0}$ into $m_{+}$ may be considered irreversible under moderate intrinsic noise changes. However, the transition to a stable $m_{-}$ is continuous, and as in Model 1, it can be reversed relatively easily. This model also belongs to the saddle-node bifurcation class and is also accompanied by a first-order phase transition. Figure 1f outlines the main difference between the bifurcation and phase transition diagrams.

### 2.2. Feedback Mechanism for Noise Regulation

The primary purpose of this work was to incorporate a feedback mechanism in Landau’s phenomenology that can effectively buffer the intrinsic noise of the system and ultimately guide stem cell populations toward a critical state. In this critical state, the system is empowered to determine its own fate through spontaneous symmetry-breaking events. To mathematically represent this process, we assumed that the evolution of $\xi_{t}$ is governed by the following equation:

(3) $$d\xi_{t}=G\left(m_{t}^{2}-M^{2}\right)dt,$$

where *G* and *M* are the control parameters of our model. Without loss of generality, we assumed M≥0. It is worth noting that this represents a negative feedback mechanism, since an increase in *m* beyond *M* results in an increase in ξ, which in turn reduces *m* (see Figure 1a,d). The system of Equations (2) and (3) is the dynamical system of interest in this work. As we will demonstrate below, this negative feedback mechanism gives rise to novel cell types.

## 3. Results and Discussion

In this section, we demonstrate the stability of the equilibrium points of the two-dimensional system described by Equations (2) and (3). Since ξ represents the strength of intrinsic noise, we will restrict our analysis to ξ≥0. Each asymptotically stable equilibrium point (SEP) corresponds to a different cell type, with pluripotent stem cells represented by SEPs at m=0 ($m_{0}$) and differentiated cells represented by SEPs at m≠0. The eigenvalues of the Jacobian matrix at the equilibrium points are $\lambda_{1,2}=\left[T\left(J\right)\pm \sqrt{T^{2}\left(J\right)-\Delta \left(J\right)}\right]/2$, where T(J) and Δ(J) are the trace and determinant of *J*, respectively. The stability of each point can then be determined using the Poincare diagram (refer to [45] for details). Moreover, if there exists an *M* such that T(J)=0 and Δ(J)>0 (non-hyperbolicity condition), the system may exhibit Hopf bifurcation, provided that the transversality and genericity conditions are also satisfied (see, for example, [46]). As we will see below, this model predicts the existence of stable limit cycles (LCs), which may be considered another possible cell type of our model.

To demonstrate spontaneous symmetry-breaking events and cell fate decisions, stability analysis is complemented by representative simulations. The numerical solution of Equations (2) and (3) was obtained using the standard Euler–Maruyama method, with an integration step of $dt=10^{-3}$. Distributions of the order parameter *m* were obtained over 1000 independent stochastic trajectories.

### 3.1. Model 1

The model exhibits two equilibrium points $E_{\pm }=\left(m_{\pm }^{*},\xi^{*}\right)=\left(\pm M,1-M^{2}\right)$, which, due to symmetry, have the same stability. The positiveness of the intrinsic noise imposes the restriction M<1. The Jacobian of the system is given by

(4) $$J=\left[\begin{array}{cc} 1-\xi -3m^{2} & -m\\ 2Gm & 0 \end{array}\right].$$

**Figure 2:**
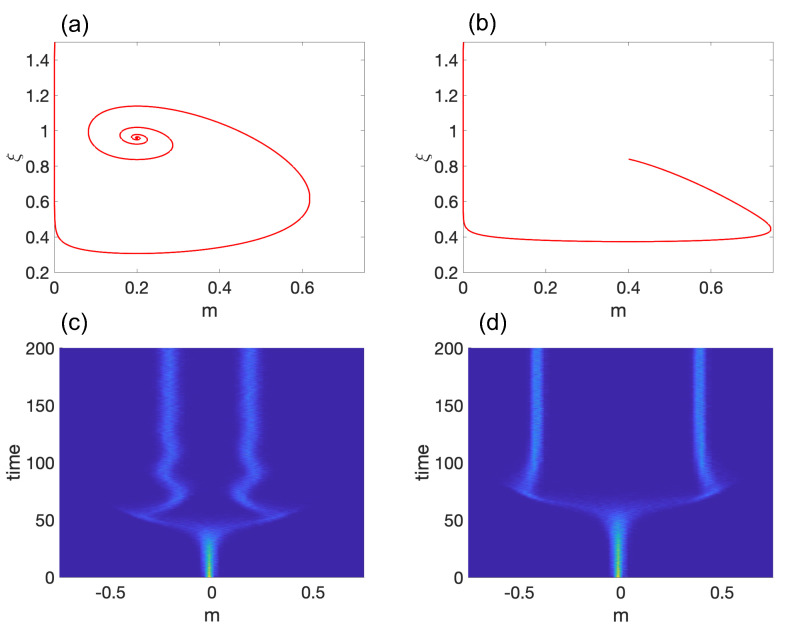
Model 1: (**a**,**b**) show representative trajectories for a stable spiral and a stable node, respectively. The corresponding probability density functions are displayed in (**c**,**d**), with light blue color indicating a high probability density. The parameter values used are M=0.2 and G=0.6 in (**a**,**c**) and M=0.4 and G=0.07 in (**b**,**d**). For (**c**,**d**), we set $\sigma =10^{-4}$.

Note that $T\left(J_{E_{\pm }}\right)=-2M^{2}$ is always negative and $\Delta \left(J_{E_{\pm }}\right)=2GM^{2}$ is always positive. Thus, both $E_{\pm }$ are asymptotically stable. Depending on the value of *G*, the equilibrium solutions can be a stable spiral (for $G>M^{2}/2$), a stable node (for $G<M^{2}/2$), or a stable degenerate node (for $G=M^{2}/2$). Hence, $\xi^{*}$ is always less than $\xi_{c}$, and as shown in Figure 2a (stable spiral) and Figure 2b (stable node), the stem cells will inevitably differentiate into either of the $E_{\pm }$ cell types.

To demonstrate self-tuned symmetry breaking in the system, we conducted 1000 independent simulations with initial conditions of m(0)=0 (pluripotent state), ξ(0)=1.5 (high noise limit), and $\sigma =10^{-4}$. Figure 2c,d show the time evolution of the probability density function (pdf), P(m). The results indicate that the system spends a significant amount of time in the pluripotent state before spontaneously differentiating and settling at $E_{\pm }$. One may reconstruct the Waddington epigenetic landscape by computing the quasi-potential $\Phi (m_{t})$∼$-\ln\left[P\left(m_{t}\right)\right]$ vs. time.

### 3.2. Model 2

The equilibria for this model are $E_{\pm }=\left(m_{\pm }^{*},\xi^{*}\right)=\left(\pm M,1+M^{2}-M^{4}\right)$, which are also symmetric and have the same stability. As in the previous model, we imposed the restriction $M<\sqrt{0.5(1+\sqrt{5})}\approx 1.272$ to ensure the positivity of the intrinsic noise. In this case, the Jacobian reads

(5) $$J=\left[\begin{array}{cc} 1-\xi +3m^{2}-5m^{4} & -m\\ 2Gm & 0 \end{array}\right].$$

Interestingly, this model exhibits a supercritical Hopf bifurcation. Note that the only instance where $T\left(J_{E_{\pm }}\right)=2M^{2}-4M^{4}$ vanishes and $\Delta \left(J_{E_{\pm }}\right)=2GM^{2}$ is positive is when $M_{c}=\sqrt{2}/2$. Moreover, both the transversality and genericity conditions are satisfied in a manner that the system displays a stable limit cycle. To summarize, for $M<M_{c}$, $E_{\pm }$ correspond to stable LCs. At $M=M_{c}$, the system undergoes a supercritical Hopf bifurcation, and $E_{\pm }$ transform into two stable fixed points. Specifically, for $M_{c}<M<1$ and M<M<1.272, $E_{\pm }$ represent a stable spiral and stable node, respectively. In our analogy, the supercritical Hopf bifurcation converts two types of cells (LCs) into two other distinctly different types of cells (SEPs).

**Figure 3:**
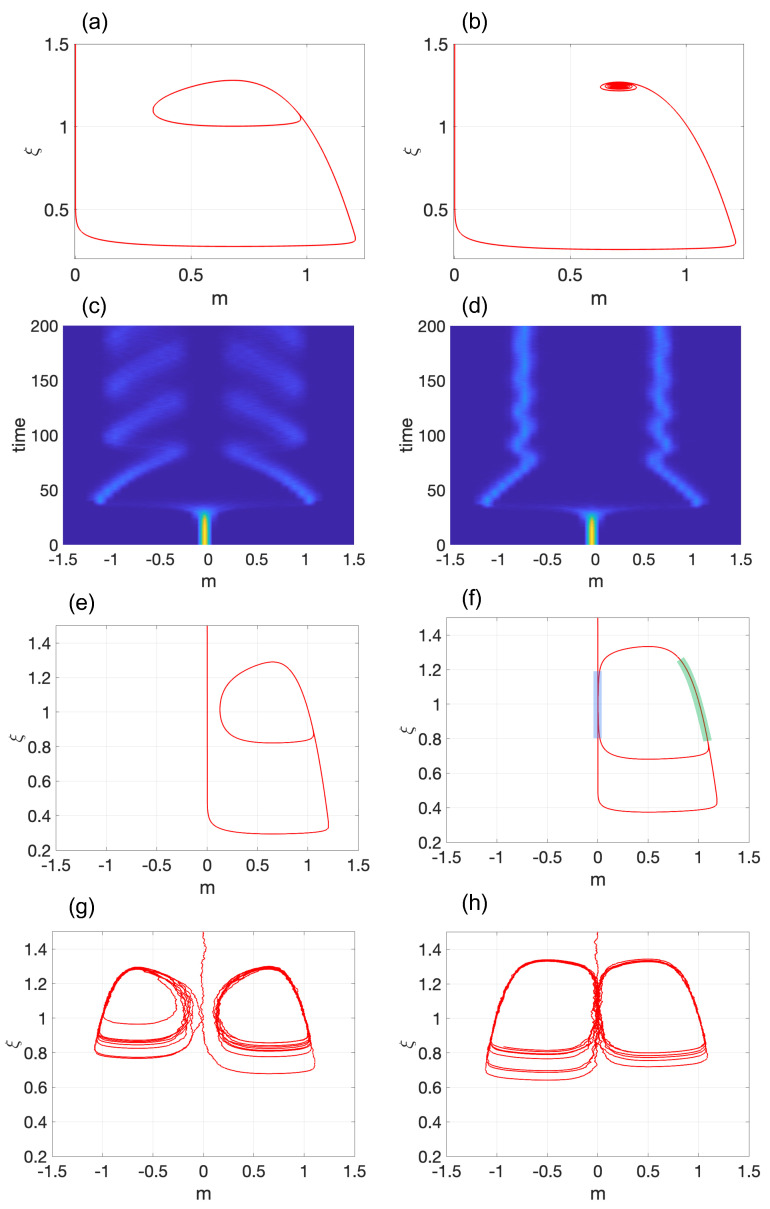
Demonstration of Hopf bifurcation in Model 2: Transition from (**a**) a stable limit cycle for $M<M_{c}$ to (**b**) a stable spiral for $M>M_{c}$. The corresponding probability densities are displayed in (**c**,**d**), respectively, with $\sigma =10^{-4}$. (**e**,**f**) depict a limit cycle with an elevated amplitude. In (**f**), the blue and green thick lines represent two slow modes at $m_{0}$ and $m\approx m_{+}$, respectively. A single stochastic simulation for (**e**,**f**) is shown in (**g**,**h**), respectively, with $\sigma =2\times 10^{-4}$. The parameter values used are M=0.68 in (**a**,**c**); M=0.71 in (**b**,**d**); M=0.65 in (**e**,**g**); and M=0.5 (**f**,**h**). In (**a**–**h**), G=0.05.

Figure 3a,b demonstrate the Hopf bifurcation for $E_{+}$ with G=0.05. Both start from a pluripotent state, m(0)=0.001, and a high internal noise of ξ=1.5. In Figure 3a, the system exhibits a stable counterclockwise limit cycle for $M=0.68<M_{c}$, and it transitions to a stable spiral for $M=0.71>M_{c}$ (Figure 3b). If the simulation had started with m(0)=−0.001, the clockwise stable LC or stable spiral for $E_{-}$ would have been observed.

Figure 3c,d present the probability density function (pdf) for $M=0.68<M_{c}$ and $M=0.71>M_{c}$, respectively, and $\sigma =10^{-4}$. Both simulations start with m(0)=0 and ξ=1.5. Similar to Model 1, the system spends a considerable time in the pluripotent state before differentiating into either the clockwise or counterclockwise LC (Figure 3b) and the left or right SEP (Figure 3d) with equal probability.

It should be noted that, if the amplitude of the LC is elevated (Figure 3e) and σ is strong enough, direct transitions from one LC cycle to another may occur (Figure 3g). This situation is known as noise-induced phenotype switching (see, for instance, [42]). When the amplitude of the LC is considerably increased, an interesting scenario arises. Figure 3f depicts an LC that periodically approaches the pluripotent state. This simulation was conducted with M=0.5 and G=0.05. It is evident that there are two modes in this case. Firstly, we see two slow modes: one at the pluripotent state (highlighted in blue) and another at a differentiated state (highlighted in green). Secondly, there are two fast modes when the trajectory quickly transitions between the pluripotent and differentiated state. Practically, the system continuously resembles the hysteresis loop between $m_{0}$ and $m_{+}$ illustrated in Figure 1e. It can be argued that this LC is a combination of the pluripotent stem cell ($m_{0}$) and a differentiated cell ($m_{+}$). With the appropriate initial conditions, it is possible to observe the LC oscillating between $m_{0}$ and $m_{-}$, as well. This scenario becomes even more intriguing when finite-size effects are taken into consideration. In this case, as depicted in Figure 3h, the system stochastically transitions between $m_{0}$ and $m_{\pm }$. As a result, a hybrid state of three cells is formed.

### 3.3. Model 3

This model exhibits two *asymmetric* equilibrium states $E_{\pm }=\left(m_{\pm }^{*},\xi_{\pm }^{*}\right)=\left(\pm M,1\pm M-M^{2}\right)$. To maintain positive ξ, $M<-0.5+\sqrt{5}/2\approx 0.618$. Here, the Jacobian is

(6) $$J=\left[\begin{array}{cc} 1-\xi +2m-3m^{2} & -m\\ 2Gm & 0 \end{array}\right].$$

Let us first analyze the stability of $E_{+}$. At $M_{c}=1/2$, we have $T(J_{E_{+}})=0$ and $\Delta (J_{E_{+}})>0$, and both the transversality and genericity conditions are satisfied. Thus, this system also exhibits a stable LC for $M<M_{c}$. However, at $M=M_{c}$, the system experiences a supercritical Hopf bifurcation, causing the LC to turn into an SEP. More specifically, for $M_{c}<M<1$, $E_{+}$ represents a stable spiral and for 1<M<1.272 a stable node. Similar to Model 2, this bifurcation can be thought of as transforming one cell type (LCs) into another cell type (SEP). For the second equilibrium point $E_{-}$, we note that for M<0.618, both eigenvalues are negative, resulting in a stable equilibrium point. In general, according to this model, a pluripotent stem cell can spontaneously differentiate into two asymmetric cell types $E_{\pm }$.

**Figure 4:**
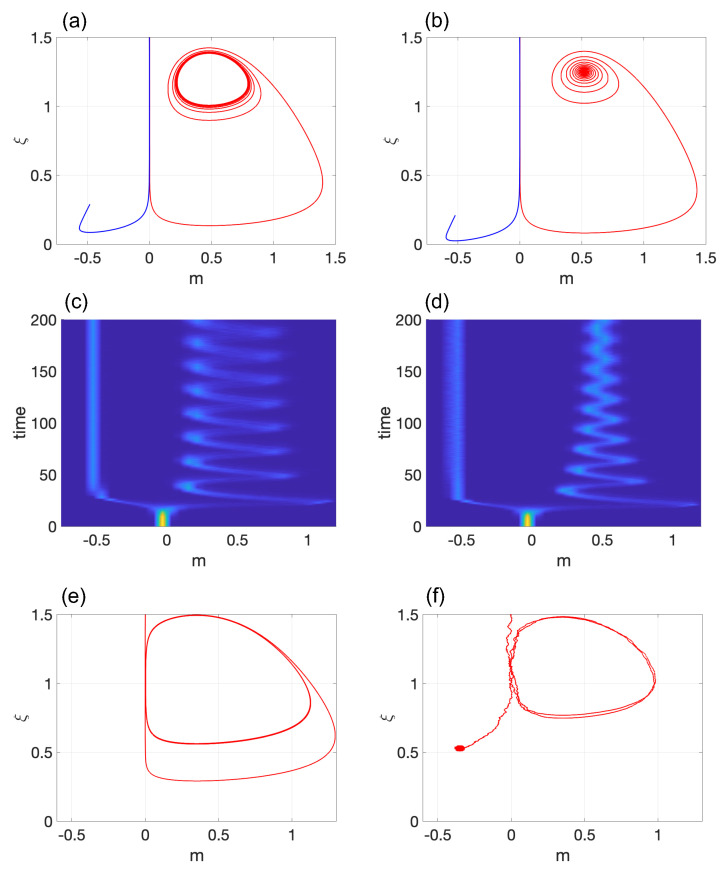
Hopf bifurcation in Model 3: Transition from (**a**) a stable limit cycle for $M<M_{c}$ to (**b**) a stable spiral for $M>M_{c}$. The corresponding probability densities are shown in (**c**,**d**), respectively, with $\sigma =10^{-4}$. Subfigure (**e**) shows a limit cycle with elevated amplitude. The corresponding stochastic simulations for (**e**) are presented in (**f**) with $\sigma =2\times 10^{-4}$. The values of the parameters are M=0.48 and (**a**,**c**); M=0.52 in (**b**,**d**); and M=0.35 in (**e**,**f**). In all subfigures, G=0.2.

The stability analysis of this model is illustrated in Figure 4. In particular, Figure 4b depicts the Hopf bifurcation for $E_{+}$ as we adjust the control parameter *M* from $0.48<M_{c}$ to $0.52>M_{c}$, respectively. It is important to note that the stability of $E_{-}$ is not affected despite changes in *M*.

The corresponding probability densities are displayed in Figure 4c,d with $\sigma =10^{-4}$. The system was initialized with m(0)=0 and ξ=1.5. Similar to Models 1 and 2, the system remains in the pluripotent state for a period of time before eventually differentiating into $E_{\pm }$. Increasing the amplitude of the LC yielded qualitatively different results compared to Model 2. If fluctuations due to finite-size effects are ignored, the system continues to switch quickly between two slow modes that represent $m_{0}$ and $m_{+}$, as demonstrated in the hysteresis loop of Figure 1e. Therefore, for large systems, the system can function in a hybrid state of $m_{0}$ and $m_{+}$. Note that the duration of time during which the system is in proximity to $m_{+}$ is notably shorter than the time it spends close to $m_{0}$. In small systems where random fluctuations cannot be ignored, the system inevitably differentiates into cell type $E_{-}$ (see Figure 4f). The reason for this is straightforward: once the system approaches an asymptotic stable equilibrium point, it cannot return to the pluripotent state. This outcome is due to the asymmetry between the two equilibrium points presented in this model.

## 4. Conclusions

This work was based on three central hypotheses. Firstly, we assumed that pluripotent stem cells operate in a state of high symmetry that enables them to co-express a certain number of genes that are found in all of the subsequent progenitors. Conversely, progenitors can only express a limited number of those genes, and thus, they assume a state of lower symmetry. Similar symmetry breaking was hypothesized for the differentiation of the progenitors into committed cells. Therefore, cell differentiation can be viewed as a *sequence* of symmetry-breaking events. Secondly, we hypothesized that pluripotency is a collective property that emerges within stem cell populations. Thirdly, we postulated that the system possesses an internal mechanism that regulates transcriptional noise and drives the system through a critical state, allowing for spontaneous cell fate decisions. These three hypotheses allowed us to draw an analogy from statistical mechanics and assume that stem cell differentiation corresponds to symmetry-breaking events arising from a series of disorder–order phase transitions. Accordingly, disorder (or high-entropy states) represents pluripotent stem cells, while order (or low-entropy states) corresponds to differentiated stem cells.

To effectively model these hypotheses, we expanded upon Landau’s mean-field theory of phase transitions, integrating a feedback mechanism to self-regulate the intrinsic noise of the system. Our methodology for investigating the dynamics of stem cell populations has the advantage of being simpler compared to other, more realistic models [25,26,27,28,29,39]. Moreover, as is the case with all mean-field models, it offers a straightforward way of understanding the underlying phenomenology of cell differentiation in terms of phase transitions. Finite-size effects were also incorporated by introducing a stochastic term, the strength of which is inversely proportional to the system size. Stability analysis of the two-dimensional dynamical system revealed the presence of several attractors (sinks and limit cycle) that correspond to different cell types. Of greater significance, the feedback mechanism has the capacity to navigate the system through various bifurcation points, thereby generating new attractors (differentiation) or switching attractors (phenotypic switch). Depending on the magnitude of the finite-size effects, stem cell populations may exist in hybrid states that encompass both pluripotent and differentiated cell types. Interestingly, our model exhibited a Hopf bifurcation, which can potentially describe the spontaneous emergence of collective oscillatory behavior in stem cell populations.

Several future directions can be explored to expand our understanding of the suggested model. One of these is to conduct a more comprehensive analysis of the effects of random fluctuations on the stability of the attractors [47]. This project could involve investigating the existence of stochastic bistability and phenotypic switching [44]. A key question in this direction would be determining whether external noise bursts could trigger cell reprogramming into a pluripotent state [4]. Another promising avenue is to extend our model beyond the standard mean-field approximation by incorporating spatial dependence in the form of stochastic partial differential equations. This approach could potentially describe self-organized criticality, as demonstrated in [48,49]. It would be interesting to explore the applicability of this approach to self-organized criticality in gene expression dynamics [50,51,52,53].

## Data Availability

Not applicable.
